# Public health ethics, through the eyes of a philosopher

**DOI:** 10.1093/pubmed/fdaa007

**Published:** 2020-04-06

**Authors:** James Wilson

**Affiliations:** Department of Philosophy, UCL, London, UK

**Public health ethics, through the eyes of a philosopher**

Bruce Laurence^1^ provides a very helpful insight into the ethical challenges that arise each day for Directors of Public Health (DsPH). While public health always involves ethical choices, the choices that DsPH must make have become significantly more challenging since 2013 when most of the frontline public health functions moved into local councils. These changes coincided with swingeing cuts in central government funding to local authorities, and so the net effect is that many councils are struggling to fund core services. Cost-effective preventive services are being squeezed out by urgent acute spending—urgent spending that in some cases will be required precisely because of the inability to fund preventive services.

Laurence writes engagingly and authoritatively about the ways that the DPH role requires grappling not just with ethical challenges within public health but also how to weigh public health against equally important non-health activities. As a philosopher who specializes in public health ethics, this is an enormously helpful insight into the kinds of ethical challenges that occur day to day for DsPH. The essay contains many more questions than answers—and in this brief response, my aim is to consider some of the ways that public health ethics could help.

Perhaps the deepest question raised by Laurence’s article is what is ethics for? He begins with the worry that an ethical framework such as the ‘four principles’ may illuminate ethical problems, but will never do the ‘heavy lifting of coming up with any clear answer’.^1^ The implication would seem to be that when it comes to day-to-day problems of public health practice, principles are of limited help.

I think that this is a bit hasty. First, the thought that if an ethical decision is difficult, it will remain difficult even once decision-makers are given access to a relevant ethical framework looks too quick. There may be decisions that look difficult until they are addressed within the right framework but then can be seen to be fairly straightforward. For example, the medical ethics literature of 40 years ago treated doctors’ ethical duties in respect of adult Jehovah’s Witnesses who lucidly wish to refuse blood transfusions as much more difficult to determine than would now be assumed. In large part this shift resulted from a clarification of the scope and content of duties of beneficence and nonmaleficence in healthcare: a consensus emerged that what counts as a benefit or a harm for the patient should be determined from the patient’s rather than the doctor’s perspective. What looks like a difficult problem may not remain one once fully considered. Even if some ethical problems remain genuinely intractable, there is a value in correctly identifying these, and not giving up too early.

Second, the developing ethical literature on public health (e.g. as represented by the journal *Public Health Ethics*) takes the development and testing of ethical frameworks to be only one element of its remit. Much of the literature is significantly more specific—focusing either on clarifying the ethical implications of particular concepts that are of core relevance for public health or examining ethical issues raised by specific interventions or policies. Work in the former category would include, for example, examining the extent to which core public health policies should be classed as paternalistic and even if they are paternalistic, whether this presents a strong reason against adopting them.^2^ Work in the latter category would include analyses of how pre-exposure prophylaxis (PrEP) should best be integrated into a safe sex ethics framework,^3^ or the ethical justifiability of minimum unit pricing for alcohol.^4^ This more specific work in turn feeds back into the further specification of ethical frameworks for public health. Overall, the best model for understanding progress in public health ethics might be the common law: a gradual accretion of larger-scale insights bottom-up from a series of smaller insights, rather than a top-down ‘engineering model’.^5^

Laurence is of course very well aware of the usefulness of case studies and calls for more of them. But the fact that, despite having a deep interest in ethics, he seems not to be aware of the extent of the existing literature in public health ethics suggests that there is much work to be done from those within the public health ethics community in making their work more accessible to those who could benefit from it. I welcome Laurence’s call for building public health ethics into training and professional development of public health workers—and I think we would both applaud the steps currently being made in this direction by the Faculty of Public Health’s public health ethics work to define professional competencies in public health ethics and law.^6^
